# Reliability of Different Dynamometers Used to Measure Isometric Shoulder Strength: A Comparative Study of Two Dynamometers in 526 Adults

**DOI:** 10.7759/cureus.97272

**Published:** 2025-11-19

**Authors:** Stefan Loske, Emma Carlisle, Ruby Strauss, Elie Walsh, Anna Gore, David Sullivan, Phillip Duke, Mark Ross

**Affiliations:** 1 Research, Brisbane Hand and Upper Limb Research Institute, Brisbane, AUS; 2 Department of Orthopaedics, Princess Alexandra Hospital, Brisbane, AUS; 3 Faculty of Medicine, The University Of Queensland, Brisbane, AUS

**Keywords:** chatillon, constant score, dynamometer, ido isometer, isometric strength, shoulder abduction strength

## Abstract

Background*:* Several devices are available to record isometric shoulder strength; however, minimal information regarding inter-device reliability and agreement is available. The objective of this study was to determine inter-device reliability and agreement of two dynamometers for measuring shoulder abduction strength.

Methods*:*Five hundred and twenty-six adults had their bilateral isometric 90^o^ shoulder abduction strength recorded with both the Chatillon^TM^ force gauge and IDO^TM^ Isometer devices by 10 independent raters. Intra-class correlation coefficients (ICCs), 95% limits of agreement (95% LoA), and minimum detectable change were calculated to determine inter-device reliability and agreement. Inter-rater and intra-rater reliability was measured in a small cohort to exclude this being a factor reducing validity.

Results*:*Inter-device reliability was excellent for both devices with an ICC of 0.89 (0.87-0.91). There is a significant positive bias with the Chatillon^TM^ force gauge measuring a mean of 0.62 kg (95% confidence interval (CI) 0.47 - 0.77 kg) more than the IDO^TM^ device. Inter-device limits of agreement were found to be approximately 50% of mean strength measurement, being dependent on the strength of the individual, with increasing inter-device error as strength increases. Inter-rater reliability was excellent for both devices, with Chatillon^TM^ achieving a superior ICC and a narrower 95% CI with 0.92 (0.80-0.97) compared to IDO^TM^ with 0.85 (0.05-0.96). Intra-rater reliability was excellent with both the Chatillon^TM ^and IDO^TM^ devices, with an ICC of 0.96 (95% CI 0.91 - 0.99) and 0.95 (95% CI 0.88 - 0.98), respectively.

Conclusions*: *With high inter-device reliability, either individual device is suitable for measuring and comparing shoulder abduction strength longitudinally. However, consistency in assessors and device use is recommended, as device measurement error may be statistically significant and may exceed minimal clinical differences for certain scores. As a consequence, care must be taken when comparing published results on shoulder abduction strength, due to variation not only in device measurement error, but also in study protocols in general.

## Introduction

The unique anatomy and biomechanical properties of the shoulder joint complex and its articulations make objective assessment of the shoulder very challenging. It is common practice for clinicians to use shoulder strength as an objective measure to assess the effectiveness of an intervention. Shoulder strength can be assessed in several different ways, evaluating the isometric, isotonic and isokinetic strength of the shoulder [1]. Isometric shoulder strength testing is performed to assess the ability of the patient to maintain a degree of tension in the shoulder muscle against a force or resistance whilst retaining static muscle contraction [2]. This type of testing has been regarded in the literature as more cost effective than isokinetic testing and an efficient way for clinicians to assess their patient’s shoulder strength [3]. Several devices have been assessed in the literature to measure isometric shoulder strength, including fixed or free spring balances and fixed or hand-held dynamometers, with good inter-rater and intra-rater reliability reported [4-11]. Few studies have assessed inter-device reliability and agreement [4,7,9-12], which is important to determine whether strength measures can be compared when two different devices are used [12]. This is particularly relevant when comparing research results on shoulder strength as a single item, or in the context of clinical scores.

A good example is the Constant score, which is an outcome measure for the shoulder first described by Constant and Murley in 1987 [13], consisting of both self-reported and performance-based components and incorporating the measurement of the shoulder strength. It is widely used, well recognised, validated and reliable outcome measure [14,15]; the Constant score is a 100-point scoring system divided into four sub-scales which include: Pain (15 points), Activities of daily living (20 points), Range of motion (40 points), and Strength (25 points). Shoulder strength measurement for the Constant score has been described as the maximal isometric abduction force calculated from the average of three maximum efforts. Participants who do not achieve 90° abduction are given a score of 0.

The method of assessment was standardised by Bankes (1998) following criticism about the lack of standardisation in performing this aspect of the test [16,17]. Constant et al. later published a review paper (2008) with modifications and guidelines for the Constant score [14]. They proposed that for greater reliability, the measurement should be performed in a standardised position, using either an Isobex^TM^ dynamometer or a spring balance technique. 

Current standard practice in our unit is the use of the Chatillon^TM^ DFX II Series 200 Dynamometer (Ametek, Florida, USA) to measure shoulder strength. Ametek specialises in strength measurements, with a range of force gauge devices available; the stated model presents a measurement accuracy of $\pm 0.3$% full scale. A standardised assessment method is used where the device is held by the assessor and applies a ‘pull force’ to resist the assessor. The Chatillon^TM^ dynamometer, however, has certain limitations, with some difficulty encountered with the stabilisation of the device when measuring stronger patients due to its handheld nature.

A newer strength gauge called the IDO^TM^ Isometer (Innovative Design Orthopaedics, London, UK) has been developed specifically for measuring shoulder strength to calculate the Constant score. It has the advantages of being portable and lightweight, and the ability to measure and record the mean shoulder applied load over a three-second period. This device has been used in research studies and clinical practice for calculating the Constant score, as evidenced by recent publications [18-20]. Kristensen et al. 2014 showed excellent absolute and relative inter-device reliability of the IDO^TM^ Isometer in comparison to the “gold-standard” IsoForceControl dynamometer (MDS Medical Device Solutions, Oberburg, Switzerland) [12].

The aim of this study was to investigate the inter-device reliability and agreement of the IDO^TM^ Isometer in measuring isometric shoulder strength in comparison to the Chatillon^TM^ Dynamometer.

## Materials and methods

Procedure 

Participants who met the eligibility criteria were recruited at Brisbane Private Hospital (Queensland, Australia), including staff and visitors attending with patients. The same participant cohort was assessed for a normative strength study for the Australian population [21]. Therefore, a wide age range and even sex distribution were intended. The study was approved by the local human research ethics committee, and all participants gave written informed consent.

Inclusion criteria were healthy bilateral shoulders as per Constant 2008 [14], whereby participants had no baseline pain and were able to complete their activities of daily living painlessly; 18 years and over; able to maintain an erect standing posture; and able to understand the English language adequately to comprehend the content of the information leaflet and consent form. 

Exclusion criteria were previous or current shoulder pain; significant trauma to any of their shoulders (requiring medical review); surgical or non-surgical intervention to any of their shoulders; shoulder, cervical or thoracic pathology; conditions that may affect their shoulder strength (e.g. neurological condition); having received local/general anaesthetic in the last 48 hours for any other medical procedure; or having had an alcoholic beverage in the last 24 hours. 

The measurements were performed by 10 research assistants, five of whom were qualified occupational therapists. The relatively large number of assessors was chosen to reflect the staff number and patient turnover in real-world practice of a busy research institute. Assessor training and test instructions given to participants were standardised. All assessments were performed independently by the assessors using both the Chatillon^TM^ and IDO^TM^ devices and measurements were recorded in kilogram-force.

**Figure 1:**
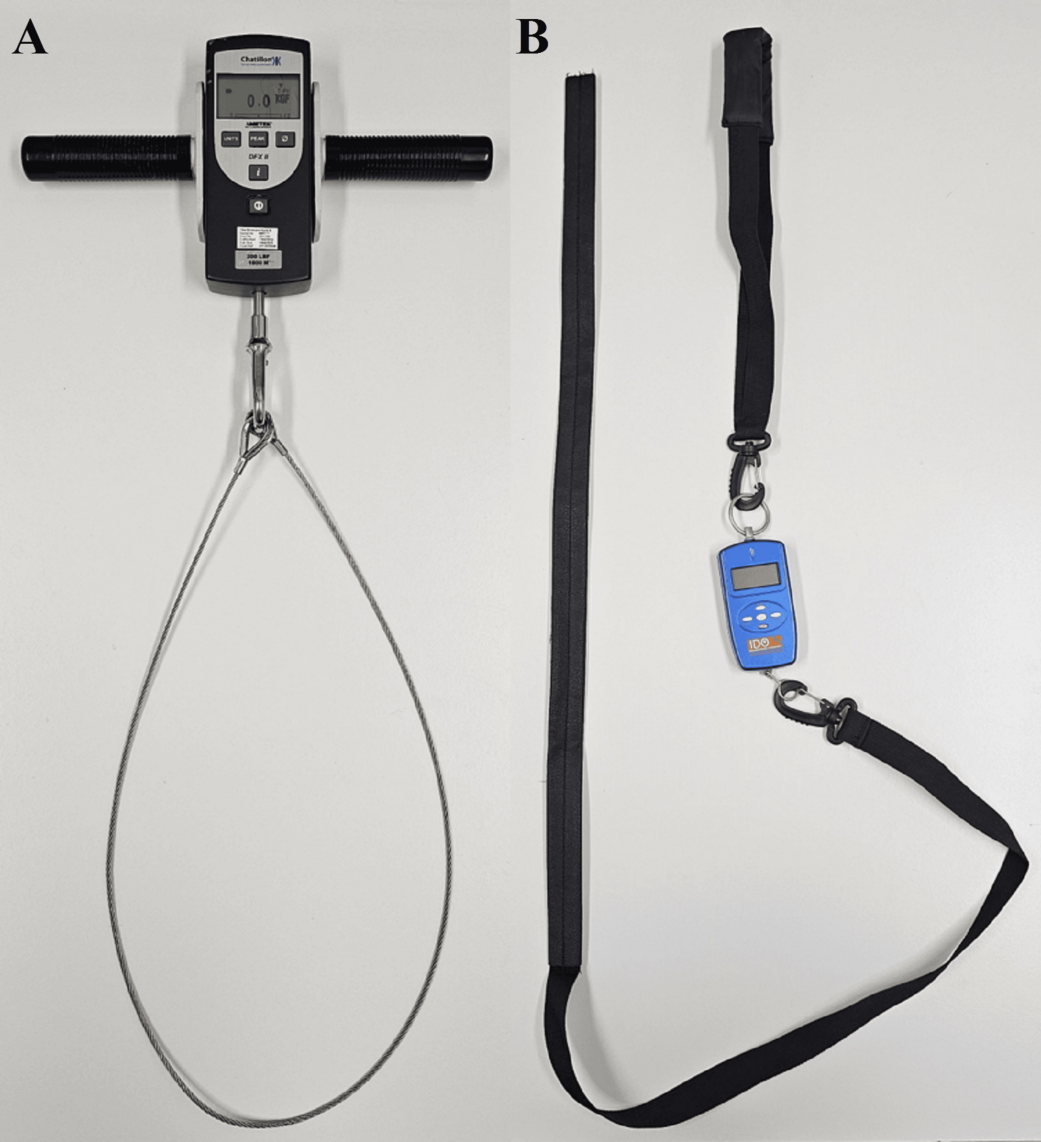
(A) The Chatillon DFX II Series 200 Dynamometer, with a handle and grasping loop. (B) The IDO Isometer digital force reader, with loop strap and long strap Chatillon^TM^ DFX II Series 200 Dynamometer: Ametek, Florida, USA; IDO^TM^ Isometer: Innovative Design Orthopaedics, London, UK

**Figure 2:**
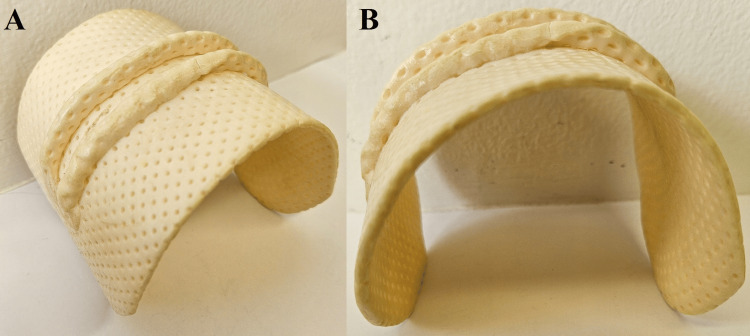
Custom thermoplastic wrist cuff used in the assessment of isometric strength using the Chatillon dynamometer

The Chatillon^TM^ DFX II Series 200 Dynamometer (Ametek, Florida, USA) is designed to objectively measure manual muscle strength and functional task requirements. It is comprised of a digital force reader where a peak force setting locks the measured result at the maximum force achieved over a three-second period for simple identification and analysis. The Chatillon^TM^ was part of an Ametek Muscle Strength Capacity Kit, paired with a handle and grasping loop (Figure 1A). A custom thermoplastic wrist cuff was designed by a qualified occupational therapist to ensure participant comfort during assessment (Figure 2).

The IDO^TM^ Isometer (Innovative Design Orthopaedics, London, UK) was designed under the guidance of Professor Ofer Levy (Reading Shoulder Unit, Brunel University) to evaluate the strength component of the Constant score (Figure 1B). It comprises a digital force reader and is specifically designed to measure and record the mean shoulder applied load, sampled over a three-second interval. Despite differences in acquisition between the Chatillon^TM^ (maximum strength) and IDO^TM^ (mean strength) devices, both are used for measuring shoulder abduction strength in clinical practice and the research setting.

The Chatillon^TM^ device was calibrated before participant enrollment began. Calibration was conducted by trained employees of a device calibration company in Brisbane (NATA Accreditation - ISO17025, SAI Global Certification - ISO9001, ISO14001, and ISO45001), who are trained to calibrate dynamometers, as per the instructions of the DFX II Series 200 dynamometer user manual. IDO^TM^ devices came with a certificate of calibration. Five IDO^TM^ devices broke during our trial and were unable to be recalibrated; the devices were not used once they had broken.

Participants were instructed to perform a warm-up session involving 10 repetitions of scapular rotation and circumduction of each shoulder with the elbow flexed. This is standard procedure to warm up the muscles in preparation for strength testing and minimise risk of injury [7,8].

**Figure 3:**
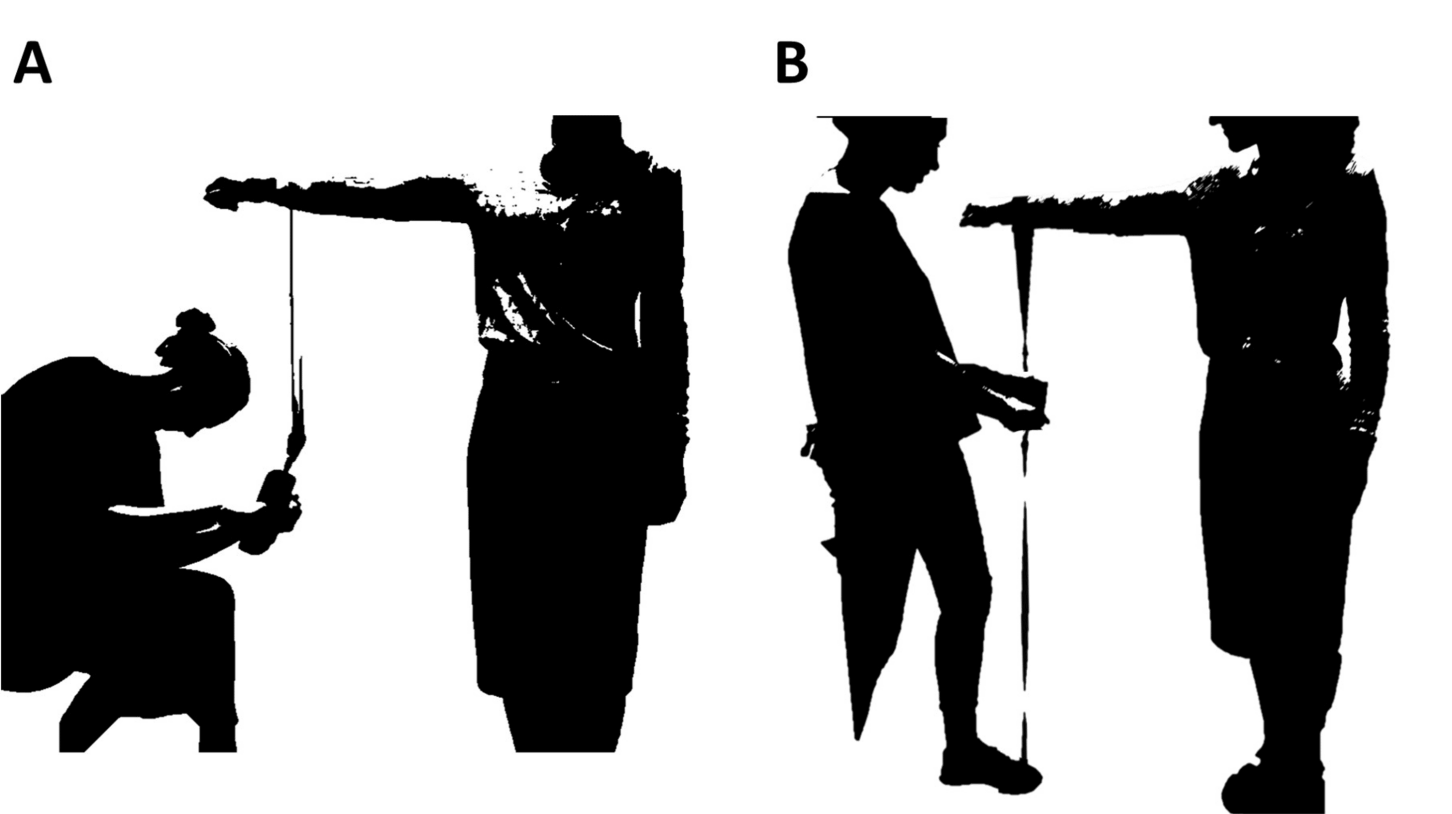
(A) Assessment of isometric strength using the Chatillon dynamometer with the handle attachment; (B) Assessment position when using the IDO isometer

Shoulder strength was assessed in the standardised position as described for the Constant score by Bankes in 1998 [16] with the participant standing erect facing forward; feet positioned shoulder width apart; arm elevated to 90° in the scapular plane; elbow held in full extension and the forearm in full pronation [22]. For the Chatillon^TM^, the assessor was seated stabilising the dynamometer using the handle attachment, and with the IDO^TM^, standing with their foot on the long strap to hold firm in the starting position (Figure 3). The loop strap used to apply the pull force was placed just proximal to the radio-carpal joint on the forearm [1]. In agreement with the findings of Hirschmann et al., the torso was not stabilised [6]. Each participant was asked to pull upwards for three seconds with a maximal effort, and the effort was resisted so as to minimise shoulder elevation.

All participants were measured on both sides by both devices, measured by the same rater across all of an individual participant's trials. The test series consisted of three successive trials of each shoulder with each device, allowing the shoulder a 1-minute rest when changing shoulder sides and devices [7]. The order of device used (Chatillon^TM^ or IDO^TM^) and shoulder side measured first was randomized using a computer-generated sequence, balancing out the participants into four groups: IDO^TM^ and left shoulder first, IDO^TM^ and right shoulder first, Chatillon^TM^ and left shoulder first, Chatillon^TM^ and right shoulder first.

Ten of the participants measured by rater 1 were re-measured by the same rater, to determine the intra-rater reliability of each device in a small sample. Another 10 participants who were measured by rater 1 were measured again by rater 2 to determine the inter-rater reliability of each device. The second rater was not present in the room during the initial assessment. Measurements were repeated after 2-3 hours in the same conditions (same environment and order of devices/shoulder sides). The purpose of conducting the intra-rater reliability tests was to determine the consistency of the raters, to ensure that each individual device’s measurements on shoulder strength were consistent and would not have an impact on the validity of the interpretation of the results. Similarly, the inter-rater reliability test was conducted to determine the consistency between raters, to ensure that assessor-related differences were minimal and would not impact the validity of the results or their interpretation. Demographic data, including sex, age, hand dominance, height, weight, occupation, and past medical history, were collected for each participant.

Statistical data analysis

The mean values for each of the three assessments were used for performing the intra-class correlation coefficient (ICC) studies. Mean and 95% confidence intervals (95% CI) were calculated. Measurement precision and absolute reliability were assessed using standard error of measurement (SEM) and calculated as standard deviation (SD) × √(1 - ICC), where SD is the standard deviation of all strength scores (mean of Chatillon^TM^ and IDO^TM^). The minimum detectable change (MDC) was calculated as 1.96 × √2 × SEM, to assess whether a change in strength between trials or devices is due to a real change or falls within the limits of error.

The Shapiro-Wilk test was used to assess for normal distribution [23]. To test for systematic bias between devices, 95% CI of mean device difference was calculated. If zero was not within the mean difference of absolute data (MDa) 95% CI, systematic bias between devices would be assumed.

To evaluate inter-device, intra-rater, and inter-rater reliability of the strength measurements, ICC was calculated with a two-way mixed effects model (absolute agreement, multiple measurements). According to Fleiss' classification, an ICC above 0.75 indicates excellent reliability; between 0.40 and 0.75, fair to good reliability; and below 0.40, poor reliability [24]. A paired t-test (Welch's two-sample t-test) was used to test for statistically significant differences, determined at p<0.05.

Bland-Altmann plots were used as a method to examine agreement between the two devices. Bland Altman plots help visualize the variability of the measurements by showing fluctuations around the mean, the magnitude of errors between devices, and to evaluate the presence of systematic errors [25]. The difference between device measurement in paired trials (y-axis) was plotted against the respective average (x-axis). Heteroscedastic data was normalised through log transformation to base 10 for the calculation of limits of agreement (LoA) as a function of the mean for easier interpretation ($-2\bar{X}(\frac{10^{a-1}}{10^{a+1}})$ and $2\bar{X}(\frac{10^{a-1}}{10^{a+1}})$, where a = 1.96 x SD_log_) (Appendix 1). Data was analysed using R Studio (Version 1.4.1106) with an alpha value of p<0.05 deemed statistically significant.

Demographic data were tabulated and presented as descriptive statistics.

## Results

**Figure 4:**
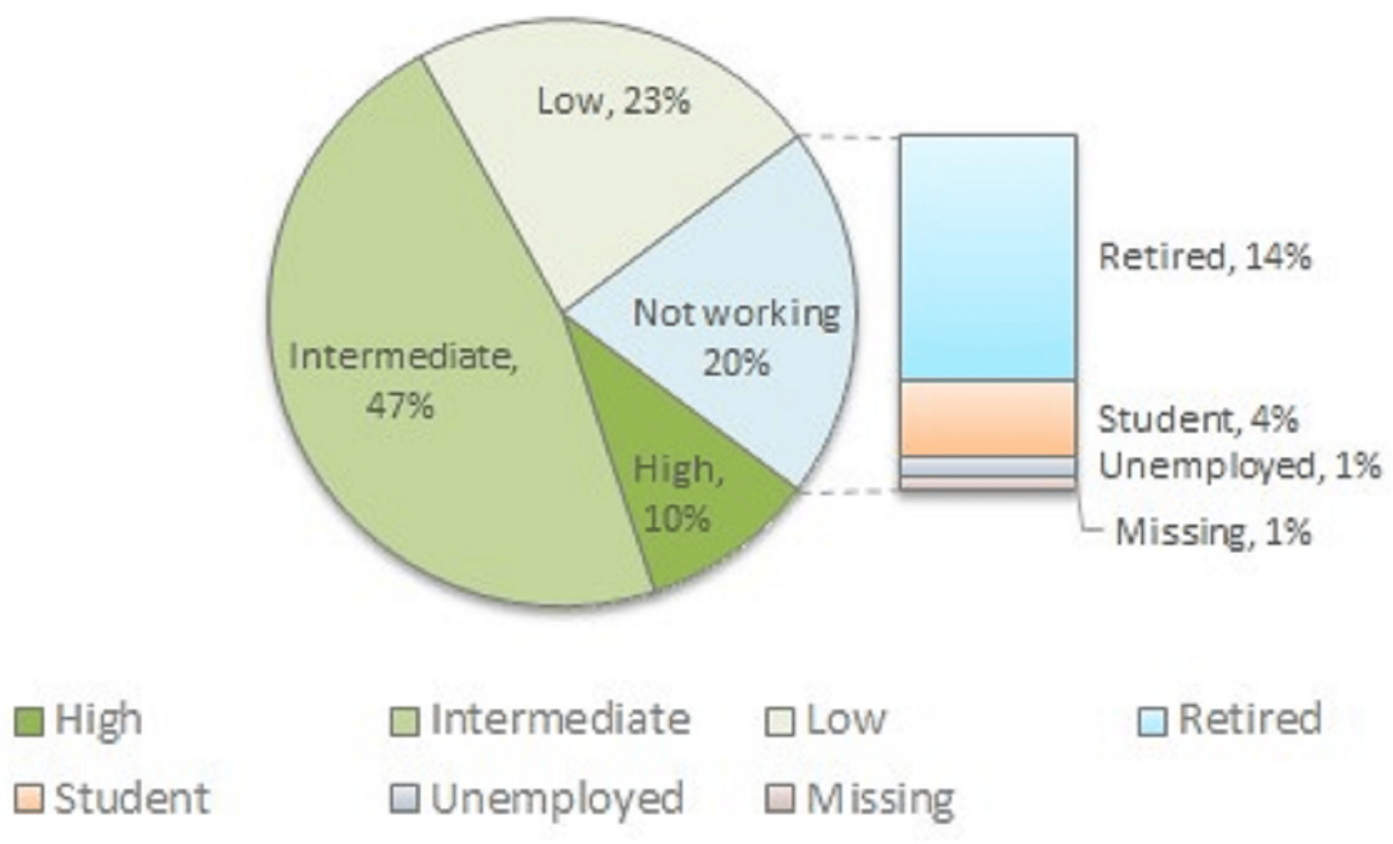
Occupational activity level of the inter-device reliability cohort (n = 526)

A total of 526 healthy volunteers (273 female participants, 253 male participants; mean ± SD: age 46.0 ± 16.54 years, height 172.28 ± 9.74 cm, weight 77.98 ± 17.61 kg) were measured by 10 raters using the Chatillon^TM^ and IDO^TM^ devices to calculate inter-device reliability and LoA. Cohort and subcohort demographics are presented in Tables 1-3 and occupational activity level in Figure 4.

**Table 1:** Descriptive statistics of inter-device reliaiblity cohort (n=526, 1052 shoulders)

Descriptive Statistics	Mean ± S.D	Min	Max
Age (years)	46.0 ± 16.54	18	89
Height (cm)	172.28 ± 9.74	145	203
Weight (kg)	77.98 ± 17.61	38	172
BMI	26.21 ± 5.47	14.49	64.74

**Table 2:** Descriptive statistics of intra-rater reliability subcohort (n=10, 20 shoulders)

Descriptive Statistics	Mean ± S.D	Min	Max
Age (years)	37.1 ± 12.58	22	61
Height (cm)	168.94 ± 8.94	152.4	181
Weight (kg)	69.53 ± 9.62	53	82
BMI	24.38 ± 3.07	19.47	30.27

**Table 3:** Descriptive statistics of inter-rater reliability subcohort (n=10, 20 shoulders)

Descriptive Statistics	Mean ± S.D	Min	Max
Age (years)	46 ± 13.22	25	61
Height (cm)	170.6 ± 6.79	163	185
Weight (kg)	70.05 ± 10.67	59	90
BMI	23.81 ± 2.59	20.28	27.72

Inter-device reliability and agreement

A correlation analysis showed inter-device reliability to be excellent at ICC = 0.89 (95% CI 0.87-0.91). The mean shoulder strength measurement with Chatillon^TM^ and IDO^TM^ was 8.72 kg, and 8.10 kg respectively (MDa = 0.62 kg), with the Chatillon^TM^ recording between 0.47 - 0.77 kg more than the IDO^TM^ (P < 0.05), with 95% confidence (Table 4). This indicated a systematic error between devices.

**Table 4:** Inter-device reliability findings (n=526, 1052 shoulders) *Values were statistically significant at p < 0.05 according to Welch's paired t-test. ICC: Intra-class correlation coefficient for two-way mixed effects model (absolute agreement, multiple measurements); LoA (limit of agreement) = MDa ± (1.96  ×  SD); SD = standard deviation of the mean; MDa: Mean difference of absolute data were calculated by subtracting Trial 2 from Trail 1; SDdif = standard deviation of the MDa; 95% CI = sample mean ± 1.96  × (SD/n); SEM (standard error of measurement) = SD × √(1 – ICC); MDC (Min detectable change) = 1.96 × √2 ×  SEM

Inter-device reliability	Device 1 (Mean±SD)	Device 2 (Mean±SD)	MDa ±Sddif (95% CI)	SEM (kg)	MDC (Kg)	ICC (95% CI)
Chatillon vs IDO	8.72± 4.06	8.10± 3.94	0.62*± 2.44 (0.47 – 0.77)	0.81	2.23	0.89 (0.87 – 0.91)
							Males	11.66±3.59	10.60±3.61	0.22*±2.71	1.17	3.24	0.81 (0.73-0.87)
Females	6.0±2.10	5.78±2.59	1.06*±2.09	1.03	2.86	0.76 (0.71-0.79)							

**Figure 5:**
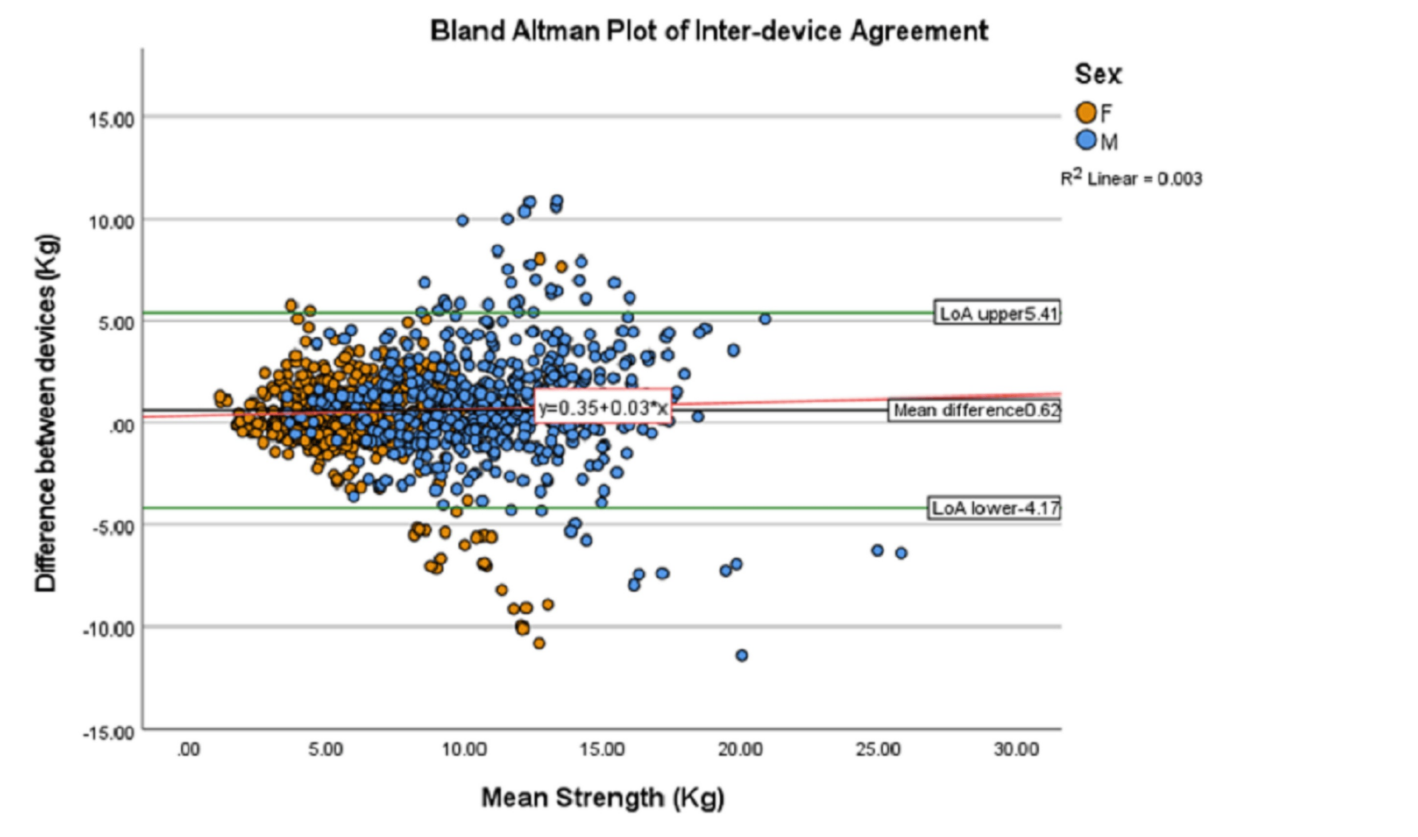
Bland–Altman graphs showing the inter-device agreement between the IDOTM and the ChatillonTM for female patients (orange) and male patients (blue). The solid green lines represent the absolute upper and lower Limits of Agreement (LoA=Bias ±1.96 x SDdif), with mean difference (bias) = 0.62

**Figure 6:**
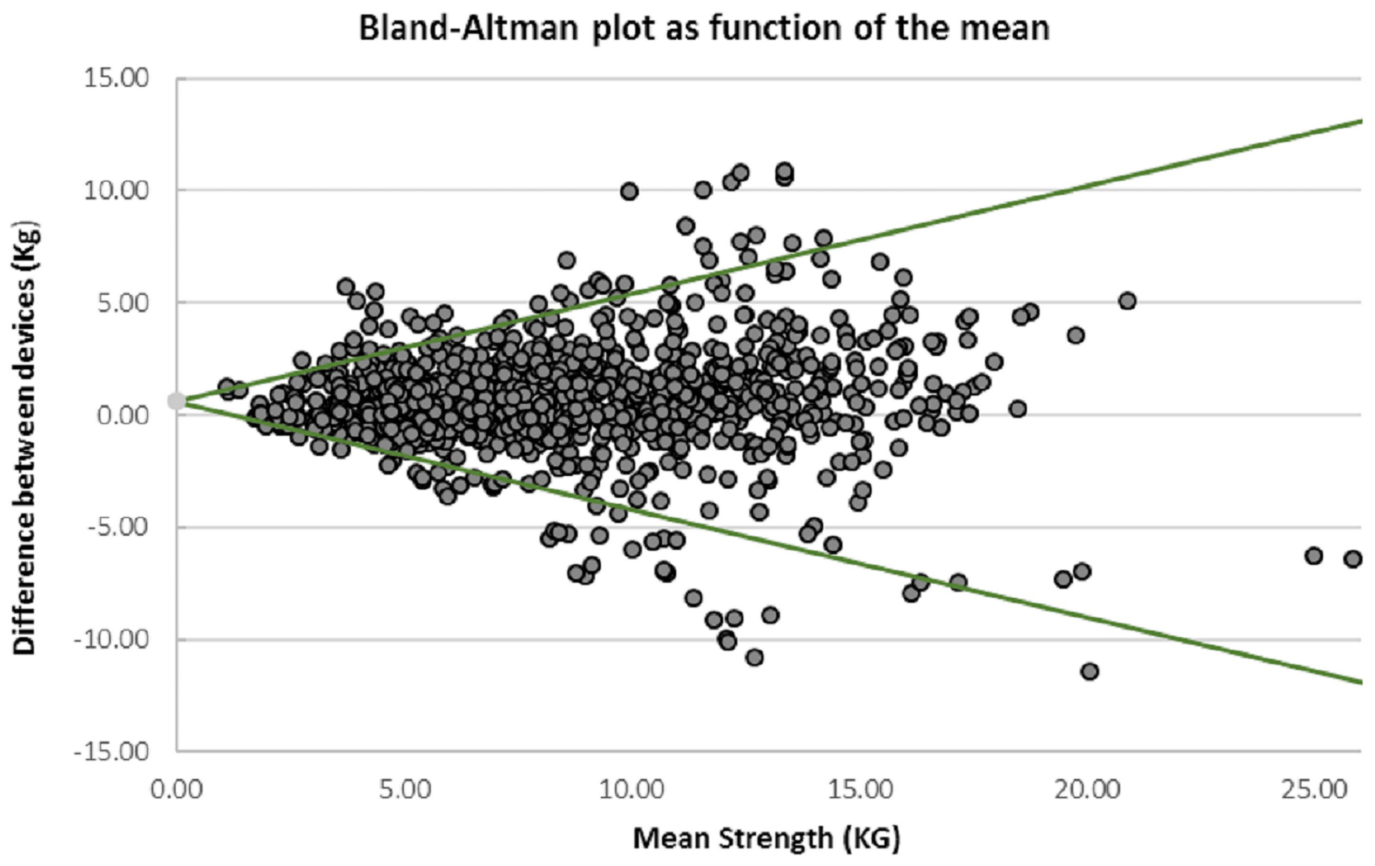
Bland–Altman plot of inter-device agreement with back-transformed LoA (green) as a function of the mean strength (0.62 ± 0.48X) LoA: Limit of agreement

The Bland-Altman plot for inter-device agreement shows an absolute 95% LoA= -4.17 to 5.41 (Figure 5). This means on 95% of occasions of measuring the same participant, the Chatillon^TM^ will be between 4.2 kg less and 5.4 kg more than the IDO^TM^ measurement. To correct for heteroskedasticity, data were then log_10_ transformed and LoA calculated as a function of the mean (Appendix 1). Device LoA can be computed for individual strength values using the function 0.62 ± 0.48X, where X is mean strength (Figure 6). For example, a patient with an average strength of 5kg measured with both devices, inter-device LoA can be calculated as -1.78 to 3.02 with 95% confidence. The LoA for a patient with 12kg shoulder strength would be -5.14 to 6.38. Where individual strength values are not available, the absolute LoA can be substituted (-4.17 to 5.41).

Inter-rater reliability

For the 10 participants measured by rater 1 and rater 2 (10 female patients; mean ± SD: age 46.0 ± 13.22 years, height 170.60 ± 6.79 cm, weight 70.05 ± 10.67 kg, BMI 23.81 ± 2.59 kg, 70% (7/10) right hand dominant, occupational activity level 70% low and 30% intermediate), inter-rater reliability was found to be excellent for both devices, with Chatillon^TM^ reaching a slightly superior ICC value and narrower 95% CI of 0.921 (0.80 - 0.97) compared to IDO^TM^ with 0.85 (0.05 - 0.96) (Table 5). 

**Table 5:** Inter-rater reliability findings (n=10, 20 shoulders) *Values were statistically significant at p < 0.05 according to Welch's paired t-test. ICC: Intra-class correlation coefficient for two-way mixed effects model (absolute agreement, multiple measurements); LoA (limit of agreement) = MDa ± (1.96  ×  SD); SD = standard deviation of the mean; MDa: Mean difference of absolute data were calculated by subtracting Trial 2 from Trail 1; SDdif = standard deviation of the MDa; 95% CI = sample mean ± 1.96  × (SD/n); SEM (standard error of measurement) = SD × √(1 – ICC); MDC (Min detectable change) = 1.96 × √2 ×  SEM

Inter-rater reliability	Rater 1 (Mean±SD)	Rater 2 (Mean±SD)	Mda ±Sddif	SEM (Kg; % of total mean)	MDC (Kg)	ICC
Chatillon	6.67± 1.77	6.41± 1.55	-0.27± 1.60	0.45 (7%)	1.25	0.92 (0.80-0.97)
							IDO	6.26± 1.25	5.43± 1.56	-0.83* ± 1.36	0.53(9%)	1.47	0.85 (0.05 – 0.96)

Intra-rater reliability

For the 10 participants measured twice by rater 1 (six female participants, four male participants; mean ± SD: age 37.1 ± 12.58 years, height 168.94 ± 8.94 cm, weight 69.53 ± 9.62 kg, BMI 24.38 ± 3.07 kg, 80% (8/10) right hand dominant, occupational activity level 50% low, 30% intermediate, 20% not working (student), intra-rater reliability was found to be excellent with both the Chatillon^TM^ and IDO^TM^ devices, with ICC of 0.96 (0.91 - 0.99) and 0.95 (0.88 - 0.98), respectively (Table 6). 

**Table 6:** Intra-rater reliability findings (n=10, 20 shoulders) *Values were statistically significant at p < 0.05 according to Welch's paired t-test. ICC: Intra-class correlation coefficient for two-way mixed effects model (absolute agreement, multiple measurements); LoA (limit of agreement) = MDa ± (1.96  ×  SD); SD = standard deviation of the mean; MDa: Mean difference of absolute data were calculated by subtracting Trial 2 from Trail 1; SDdif = standard deviation of the MDa; 95% CI = sample mean ± 1.96  × (SD/n); SEM (standard error of measurement) = SD × √(1 – ICC); MDC (Min detectable change) = 1.96 × √2 ×  SEM

Intra-rater reliability	Rater 1 first measurement (Mean±SD)	Rater 1 second measurement (Mean±SD)	Mda ±Sddif	SEM (Kg; % of total mean)	MDC (Kg)	ICC
Chatillon	8.85± 2.73	8.89± 3.18	0.04± 1.24	0.55 (6%)	1.52	0.96 (0.90-0.99)
							IDO	8.35± 2.76	8.48± 3.16	0.13± 1.29	0.55 (7%)	1.52	0.95 (0.88-0.98)

## Discussion

The statistical analysis shows excellent results for inter-device reliability, which is in agreement with the existing literature comparing shoulder abduction strength devices [7-9,11].

Despite the excellent reliability, we stress the importance of analysing the agreement when comparing devices. Reliability gives an indication of a linear relationship; however, it does not assess equality in measurements, which is an important indicator of systematic bias. Between the two devices used in this study, there was a significant positive bias with the Chatillon^TM^ measuring a mean of 0.62 kg (95% CI 0.47 - 0.77 kg) more than the IDO^TM^ device. This difference was anticipated and is likely explained by the two different modes of acquisition of the devices: The Chatillon^TM^ measures the peak strength during the three-second acquisition interval, whereas the IDO^TM^ records the mean strength during the three seconds. Inter-device LoA were found to be equal to approximately 50% of mean strength measurement (LoA=0.62±0.48X). This positive bias can be put into perspective by applying it to the Constant score. Of a total of 100 points between 14 items, the Constant score assigns approximately 2 points per 1kg of force (or 1 point per 1lb of force), of 25 total points attributed to the strength section [13]. For a patient with an average shoulder strength of 10 kg (20 points), this would result in ±10 points variation in the final calculated Constant score between devices. The minimal clinically important difference (MCID) of the Constant score in surgically treated rotator cuff patients ranges from 6.3 to 10.4 points, which is equal to or is exceeded by the effect of the potential inaccuracy of the strength measurement for individuals with average shoulder strength [26-28]. Furthermore, it must be considered that the 95% LoA is wider for stronger individuals and can influence the precision of a single measurement as described by Vermeulen et al. [11]. This illustrates the misleading interpretation of ICCs (in this instance, 0.89) in device reliability studies and underlines the relevance of considering device differences in the interpretation of published results on shoulder strength in the clinical and scientific context.

In this study, only two of many devices on the market have been compared. Our findings demonstrate the significant differences that can be found between similar devices used for the same clinical or scientific context. It remains unclear, inasmuch these findings can be generalised beyond the devices analysed within this study, but a certain awareness of this potential impacting factor in relevant contexts may be warranted.

Cohort demographics and methods of acquisition among studies assessing inter-device reliability of shoulder abduction strength vary considerably. Most studies only enrolled younger volunteers [7-9,11]. Volunteers in our study were intentionally recruited to be representative of a wide age range (18 - 89 years) and a relatively equal distribution of sexes (273 female participants, 253 male participants) due to dual participation in a study on normative shoulder abduction strength. Despite this, a large sample size with a wide range in measures can contribute to bias in ICC calculations [29]. For this reason, both reliability and agreement must be considered when comparing devices, as previously highlighted in the paragraph above.

The inter-rater reliability was significantly lower for the IDO^TM^ with a notably wider 95% CI; however, both devices meet excellent correlation requirements as described by Fleiss [24]. The discrepancy in measurement could be explained by assessor familiarity with devices; the Chatillon^TM^ device is the standard shoulder measurement used within our clinic; thus, the assessors are more accustomed to using this device. 

Intra-rater reliability was very high and supports the hypothesis that measurements by the same examiner with the same device are highly reproducible. These results not only meet the cut-off value of ICC 0.75 for excellent reliability defined by Fleiss [24], but also the higher threshold of ICC 0.9 for intra-rater reliability suggested by Schrama [30]. We can therefore conclude that intra-rater reliability has not impacted the validity of the results. All 10 volunteers enrolled in the intra-rater cohort were measured by the same examiner.

There is inconsistency in the definition of shoulder abduction, for example, in the plane of movement (coronal, scapula), in the degrees of abduction and shoulder rotation and in the position during measurement (sitting, standing) [6,11]. Even the terminology, e.g. elevation and abduction, is applied differently in the literature and can be misleading. Vermeulen et al. measured shoulder abduction strength in a sitting position, 45° shoulder abduction in the scapular plane with the elbows flexed 90° and resistance applied over the lateral epicondyle of the elbow [11], whereas shoulder elevation is defined similar to shoulder abduction in our study with the shoulder internally rotated, 90° elevated in the scapular plane, elbows extended and resistance applied over the wrist. The importance of measurement standardisation, as evidenced by Conboy et al., Bankes et al., and Rocourt et al. previously [15-17], and applying scientifically published and widely accepted measuring procedures, was a central pillar of this study to reduce potential bias [1,16].

As mentioned, both the Chatillon^TM^ and IDO^TM^ have excellent intra-rater and inter-rater reliability for the measurement of isometric shoulder abduction strength. Intra-rater and inter-rater reliability were not the primary focus of this study, thus only examined in a small sample size, with the intention to exclude an impact on the validity of the interpretation of the results. Based on the results of this study, we suggest it is most important to have a standardised measurement protocol and, where possible, a consistent device and assessor to produce reliable repeated measurements. Furthermore, care must be taken when comparing published study results that utilise different isometric shoulder strength measurement devices, due to the potential clinically significant impacts of inter-device measurement variation and error.

A difference in ICC value between male (0.81) and female (0.76) subjects was calculated. However, this difference of 0.05 between genders was seen as non-substantial, with additionally both gender-based ICC values falling within the same cut-off range specified by Fleiss [24].

We consider the sample size and demographic heterogeneity of our cohort, as well as the prospective design, to be the principal strengths of our study. To our knowledge, this is the first study assessing inter-device agreement of the Chatillon^TM^ and IDO^TM^ devices. Our results widely support the conclusion drawn by Kristensen et al. [12]. An interchangeable use of the two devices reduces the reliability of the data and should be avoided, with an error of approximately 50% to be expected when devices are interchanged. This is of most importance in scientific datasets and longitudinal follow-ups of cohorts focusing on the single item of shoulder abduction strength. The relevance of avoiding interchangeable use of these devices may extend to daily clinical practice as our results demonstrate. We deem the measurement error to potentially have a significant impact on interpreting changes in clinical outcome scores, as we have shown for the example of the Constant score, given that it meets or exceeds the listed MCIDs for rotator cuff reconstructions of 6.3 - 10.4 points. The IDO^TM^ is certainly the smaller and more portable device (100 x 50 mm and <0.2 kg vs. 190.6 x 72.6 x 41.1 mm and 0.7 kg), and also 25% less expensive than the Chatillon^TM^ device. However, five of the IDO^TM^ devices broke during our trial and were unable to be recalibrated, thus inflating the total device cost in the study.

Certain weaknesses need to be discussed as well. Enrolment took place in one private hospital, representing a population and single-centre bias. In voluntary studies with healthy patients, a responder bias can be taken into account whereby health-conscious and fitter volunteers are more likely to participate [22]. Secondly, a total of 10 assessors, heterogeneously consisting of researchers and physical therapists, contributed to the study data collection, representing another potential bias. This may be evidenced by the slightly inferior inter-rater compared to intra-rater reliability in our results. We worked to reduce this error by providing a standardised assessment protocol and regular training for the assessors. Additionally, we consider this number of assessors closer to real-world practice in a busy clinic and research institute. Finally, given that no imaging studies were conducted, asymptomatic rotator cuff diseases cannot be fully excluded, although this does not necessarily have to influence shoulder abduction strength, which was shown in Constant score measurements [19].

The decision regarding which device to use depends on individual preferences, the intended application of measurements and the practicality of the general setting of the rooms in clinical practice.

## Conclusions

With high inter-device reliability, either individual device is suitable for measuring and comparing shoulder abduction strength longitudinally. However, for clinical practice, the interchangeable use of the two devices is not recommended, due to relevant device measurement errors and the potential effect to reach or exceed the MCID for certain scores, like the Constant score in the context of rotator cuff surgery. Similarly, in a scientific context and for identifying changes in individual patient strength, an interchangeable use of the different devices must be avoided. While the generalisability of these findings beyond the devices mentioned is not fully established, an awareness of potential variation in device measurement error and differences in study protocol may be warranted when comparing published results of shoulder abduction strength as a single item or as part of scores.

## Appendices

Appendix 1

ICC: Intra-class correlation coefficient (2,k) for two-way random effects model (absolute agreement type, average).

Absolute LoA (limit of agreement) = MDa ± (1.96 x SD)

SD (standard deviation)= SD of the mean of trial 1 and trial 2.

SEM (standard error of measurement) = SD × √(1 - ICC)

MDC (Min detectable change) = 1.96 × √2 ×  SEM 

MDa: Mean difference of absolute data were calculated by subtracting Trial 2 from Trial 1.

Standard deviation of difference of log10 transformed variables (SD_log_) = 0.126 

Mean of log10 transformed variables $\bar{X}$ = 0.87

Bias (Mean difference of absolute values)= 0.60

Lower and Upper Limit of Agreement (LOA) as a function of the mean

$-2\bar{X}(\frac{10^{a-1}}{10^{a+1}})$ and $2\bar{X}(\frac{10^{a-1}}{10^{a+1}})$, where a = 1.96 x SD_log_

Where X is the average strength (absolute), Upper LOA = 0.60 + 0.48X; Lower = 0.60 - 0.48X
